# miR‐130a‐3p regulated TGF‐β1‐induced epithelial‐mesenchymal transition depends on SMAD4 in EC‐1 cells

**DOI:** 10.1002/cam4.1981

**Published:** 2019-02-11

**Authors:** Xiaokang Tian, Qian Fei, Mingyu Du, Hongming Zhu, Jinjun Ye, Luxi Qian, Zhiwei Lu, Wenjun Zhang, Yan Wang, Fanyu Peng, Jie Chen, Baoling Liu, Qian Li, Xia He, Li Yin

**Affiliations:** ^1^ Xuzhou Medical University Xuzhou Jiangsu China; ^2^ Jiangsu Cancer Hospital, Jiangsu Institue of Cancer Research, Nanjing Medical University Affiliated Cancer Hospital Nanjing Jiangsu China; ^3^ The Fourth Clinical Medical College of Nanjing Medical University Nanjing Jiangsu China

**Keywords:** Epithelial‐mesenchymal transition (EMT), Esophageal squamous cell carcinoma (ESCC), SMAD4, transforming growth factor‐β (TGF‐β)

## Abstract

Metastasis and invasion are the primary causes of malignant progression in esophageal squamous cell carcinoma (ESCC). Epithelial‐mesenchymal transition (EMT) is crucial step of acquisition of "stemness" properties in tumor cells. However, the mechanism of esophageal cancer metastasis remains unclear. This research was designed to explore the role and mechanism of SMAD4 and miR‐130a‐3p in the progression of transforming growth factor‐β (TGF‐β)‐induced EMT in vivo and in vitro. The expression of miR‐130a‐3p in ESCC cell line and normal esophageal epithelial cell was determined by RT‐qPCR. The protein expression levels of TGF‐β‐induced changes in EMT were analyzed by western blotting and immunofluorescence. Dual‐luciferase report assays were used to validate the regulation of miR‐130a‐3p‐SMAD4 axis. The effect of miR‐130a‐3p and SMAD4 in TGF‐β‐induced migration, invasion in the ESCC cell line EC‐1 was investigated by wound healing assays and Transwell assays. Here we found that knocked down SMAD4 could partially reverse TGF‐β‐induced migration, invasion, and EMT progression in the ESCC cell line EC‐1. miR‐130a‐3p, which directly targets SMAD4, is down‐regulated in ESCC. miR‐130a‐3p inhibits the migration and invasion of EC‐1 cells both in vitro and in vivo. Finally, miR‐130a‐3p inhibits TGF‐β‐induced EC‐1 cell migration, invasion, and EMT progression in a SMAD4‐dependent way. In conclusion, this study provides new insights into the mechanism underlying ESCC metastasis. The TGF‐β/miR‐130a‐3p/SMAD4 pathway could be potential targets for clinical treatment of ESCC.

## 1 INTRODUCTION

Esophageal squamous cell carcinoma (ESCC) is one of the most common malignant tumors in the digestive system worldwide. Although multimodal therapies have improved survival outcome and prognosis of ESCC, most patients were diagnosed at late stage along with regional or distant metastasis.^1^, ^2^ The poor outcome of ESCC is related to advanced TNM stages and the metastatic propensity.^3^, ^4^ Therefore, the molecular mechanism leading to tumorigenesis and tumor progression of ESCC requires further investigation.

Recently, a study has reported that epithelial‐mesenchymal transition (EMT) is associated with cancer cell invasion and metastasis.^5^ During EMT, markers of mesenchymal cell such as vimentin and N‐cadherin are upregulated, conversely, markers of epithelial cell such as β‐catenin and E‐cadherin are downregulated.^6^ Eventually, the cell polarity of epithelial tumor cells is lost,^7^ and the cells are loosely connected.^8^ At the same time, tumor cells are more susceptible to attack and metastasis.

A large number of signaling pathways participate in the EMT process of human cancer.^9^ The superfamily of transforming growth factor‐β (TGF‐β) plays a significant part in tumorigenic as well as metastatic processes of ESCC.^9^ By ligand binding to type II receptor, the cascade of TGF‐β signaling pathway is initiated. This process phosphorylates and recruits type I receptors.^10^ The intracellular effectors (SMAD2/SMAD3) are phosphorylated by the activated type I receptors, which come into being a complex with SMAD4 and are transported to the nucleus for transcriptional regulation.^11^ As a key molecule in the TGF‐β signaling pathway, SMAD4 undoubtedly plays an irreplaceable role in the TGF‐β‐mediated EMT.^12^, ^13^, ^14^ However, the mechanism of action of SMAD4 in the progression of EMT in esophageal cancer remains to be studied.

MicroRNAs (miRNAs) are noncoding RNA molecules consisting of 19‐24 nucleotides.^15^, ^16^ By binding to complementary sequences in the 3′ untranslated regions, miRNAs can function as a regulator of gene expression at the posttranscriptional level. Increasing evidence supports that diverse miRNAs participated in regulating EMT in ESCC cells.^17^ It has been informed miR‐130a‐3p was downregulated and had an inhibitory effect on many kinds of cancers, including prostate cancer,^18^ hepatocellular carcinoma,^19^ chronic granulocytic leukemia,^20^ nonsmall cell lung cancer cells, and breast cancer.^21^, ^22^ Nevertheless, the corresponding molecular mechanism and the exact role of miR‐130a‐3p in ESCC remains unsearchable.

Our study examined the role of miR‐130a‐3p in ESCC development and progression. The results suggested that miR‐130a‐3p antagonized the TGF‐β1‐mediated EMT, invasion, and migration of ESCC cells depend on SMAD4. In addition, this study provides an important cellular and molecular basis for miR‐130a‐3p as one of the probable therapeutic targets for highly aggressive ESCC.

## 2 MATERIALS AND METHODS

### 2.1 Cell lines and cell culture

Human ESCC cell lines (EC‐1, EC‐3, EC‐4, EC‐6, ECa‐109, TE‐1) were cultured in RPMI 1640 (Corning, Manassas, VA, USA) complemented with 10% fetal bovine serum (FBS, Gibco, Grand Island, USA), 100 μg/mL streptomycin, and 100 units/mL of penicillin. Normal esophageal epithelial cell line (Het‐1A) were maintained in DMEM supplemented with 10% FBS (Hy Clone, USA).

### 2.2 TGF‐β treatment

EC‐1 cell lines were starved overnight before treatment. To stimulate cells with TGF‐β1 (R&D Systems, USA), these cells were incubated with 1% FBS containing 10 ng/mL TGF‐β1 for 48 hours to reach the EMT state.^23^

### 2.3 Immunofluorescence (IF) Staining

We seeded EC‐1 cells in 24‐well culture dishes and treated with TGF‐β1 as mentioned above. After the treatment, the medium was discarded, and the EC‐1 cells were washed three times with PBS. Cells were stereotyped in PBS with 4% paraformaldehyde for 20 minutes and permeabilized in 0.5% Triton X‐100/PBS for 5 minutes at 23‐28°C. We blocked the cells with 2% bovine serum albumin in PBS for 30 minutes and then incubate the cells with anti‐E‐cadherin (1:100; Proteintech) and anti‐vimentin (1:100; Boster, Wuhan, China) antibodies at 37°C for 2 h. Finally, after washing with PBS, the cells were stained with a combination of the secondary antibodies conjugated to fluorescein isothiocyanate and tetramethyllrhodamine for 1 hour. Nuclei were labeled with 4',6‐diamidino‐2‐phenylindole and observed with the microscope (Olympus Corporation, Tokyo, Japan) at a multiple of ×200.

### 2.4 RNA isolation and quantitative real‐time PCR (qRT‐PCR) assays

Total cellular RNAs were extracted using Trizol reagent (Invitrogen, Carlsbad, CA) in light of the manufacturer's mRNA expression assay. We used ReverTra Ace qPCR RT Kit (TOYOBO, Osaka, Japan) reverse transcribe miR‐130a‐3p and measured by SYBR Green quantitative RT‐PCR (qRT‐PCR) on the ABI7500 real‐time PCR (Applied Bio‐systems). We used U6 RNA as a miRNA endogenous control. The comparative CT method (2^‐ΔΔCt^) was used for the relative expression analysis. All the experiments were performed in triplicate.

### 2.5 Cell transfection

The miR‐130a‐3p mimic and negative control (NC) mimic were purchased from RiboBio (Guangzhou, China). RiboBio (Guangzhou, China) provided SMAD4 siRNA (Si‐SMAD4) and control (Si‐NC). We subcloned the full‐length SMAD4 into the pcDNA3.1 plasmid to increase the expression level of SMAD4, and the control plasmid was pcDNA3.1 empty vector. EC‐1 cells were seeded in 6‐well plates (1.5 × 10^9^ cells per well) under the action of Lipofectamine 2000 (Invitrogen, Carlsbad, CA) conferring to the manufacturer's protocol for 6 hours. After transfection for 48 hours, the cells were collected for western blot analysis or qPCR assays.

### 2.6 Wound healing assays and Transwell assays

The cells were planted in 6‐well plates and cultured in serum‐free medium until growth was spread over the bottom of the plate. The monolayer cells were scratched with a 200 µL RNase‐free pipette; and the migration capacity was determined by the relative gap distance. At different time intervals (0 and 24 hours), the relative gap distance of cell was taken at ×200 magnification under an inverted microscope. Tumor cells’ invasive abilities in vitro were assessed by Matrigel‐coated Transwell (BD Biosciences, San Diego, CA, USA) suitable for the manufacturer's protocols. Briefly, 5 × 10^4^ cells in 200 μL of serum‐free medium were planted into the upper chamber of the 8‐10 μm pore size membrane, and a medium containing 20% FBS was added into the lower chamber. After 24 hours of incubation at 37°C in 5% CO_2_, the migrant cells that had adhered to the lower surface were fixed and stained. Under an inverted microscope, we randomly selected five cells from each well for counting, and the trial was repeated three times independently. The process for the migration assay was performed in the same manner as the invasion assay, and there was no matrix gel covering the top chamber filter.

### 2.7 Tumor xenograft model

As mentioned earlier, a brilliant experimental model of spontaneous lymph node metastasis has been found.^24^ Athymic male BALB/c nude mice (6‐8 weeks old) were obtained from Yangzhou University Medical Center (Yangzhou, China). Then, spontaneous lymph node metastasis was induced by injecting 2 × 10^6^ EC‐1 cells into the foot pad of each mouse. The nude mice were separated into three groups (n = 5 mice each) when the xenograft volumes reached 60 mm^3^, namely, NC group (EC‐1/saline buffer) as the G1 group, control argomir group (EC‐1/control argomir group) as the G2 group, and miR‐130a‐3p argomir group (EC‐1/miR‐130a‐3p argomir group) as the G3 group. Subsequently, 20 μL of saline buffer was injected subcutaneously into the footpad to the tumor mass of each mouse in the G1 group. Additionally, 5 nmol/miR‐130a‐3p argomir or control argomir (RiboBio) in 20 μL of saline buffer was injected hypodermically into the footpad, particularly into the tumor mass of each mouse in the G3 or G2 group. Three groups were administered based on the divided doses for a total period of 1 month, and the drug was injected five times for every 6 days. The experiment was completed when the control primary tumor began to invade the popliteal region or when the mouse died. The mice were euthanized 7 weeks after injection, and the tumors were weighed after autopsy. The attendance of popliteal lymph node metastases was observed by autopsy. The animal experiment was approved by the Animal Science Committee of the Animal Science of Nanjing, China.

### 2.8 Luciferase activity assay

We performed luciferase reporter assay as described before. The wild‐type and mutated fragments of the human SMAD4 3′UTR region containing the predicted binding site for miR‐130a‐3p were created and injected into the luciferase reporter vector. We co‐transfected the EC‐1 of ESCC cell lines (1.5 × 10^9^ cells per well) with the following reagents: miR‐130a‐3p mimic or NC, SMAD4‐3′UTR wild‐type vector or mutant vector containing firefly luciferase reporter, and the 3'UTR of the SMAD4 using the transfection reagent Lipofectamine 2000 (Invitrogen, Carlsbad, CA). Forty‐eight minutes later, we harvested the cells and then analyzed the luciferase activities according to the description of Dual‐Luciferase Reporter Assay System (Promega). Here, we performed the experiment in triplicates.

### 2.9 Western blot analysis

The cells were extracted using modified RIPA (RIPA Lysis buffer) buffer (Beyotime, Shanghai, China) after 48 hours of transfection. Then, equal amounts of protein (20 μg) concentration were quantified with a BCA protein assay kit (Beyotime, Shanghai, China). Fifteen‐microliter of protein from each sample was subjected to SDS‐PAGE and transferred to polyvinylidene fluoride (PVDF) membranes (Millipore, Bedford, MA, USA). The PVDF membranes were incubated with the indicated primary antibodies, followed by HRP‐linked secondary antibodies. Immunoreactive bands were imaged with ECL detection reagent (Millipore, Billerica, MA, USA). All data analyses were repeated three times independently.

### 2.10 Statistical analysis

Results shown in this study were illustrated as the mean ± standard deviation of at least three independent experiments. We performed statistical analysis using the Student's *t* test or analysis of variance (ANOVA) on the functional studies section using GraphPad Prism 5.0 and SPSS 13.0 software. A *P *< 0.05 was considered to be statistically significant.

## 3 RESULTS

### 3.1 TGF‐β induces EMT in ESCC cells

**Figure 1:**
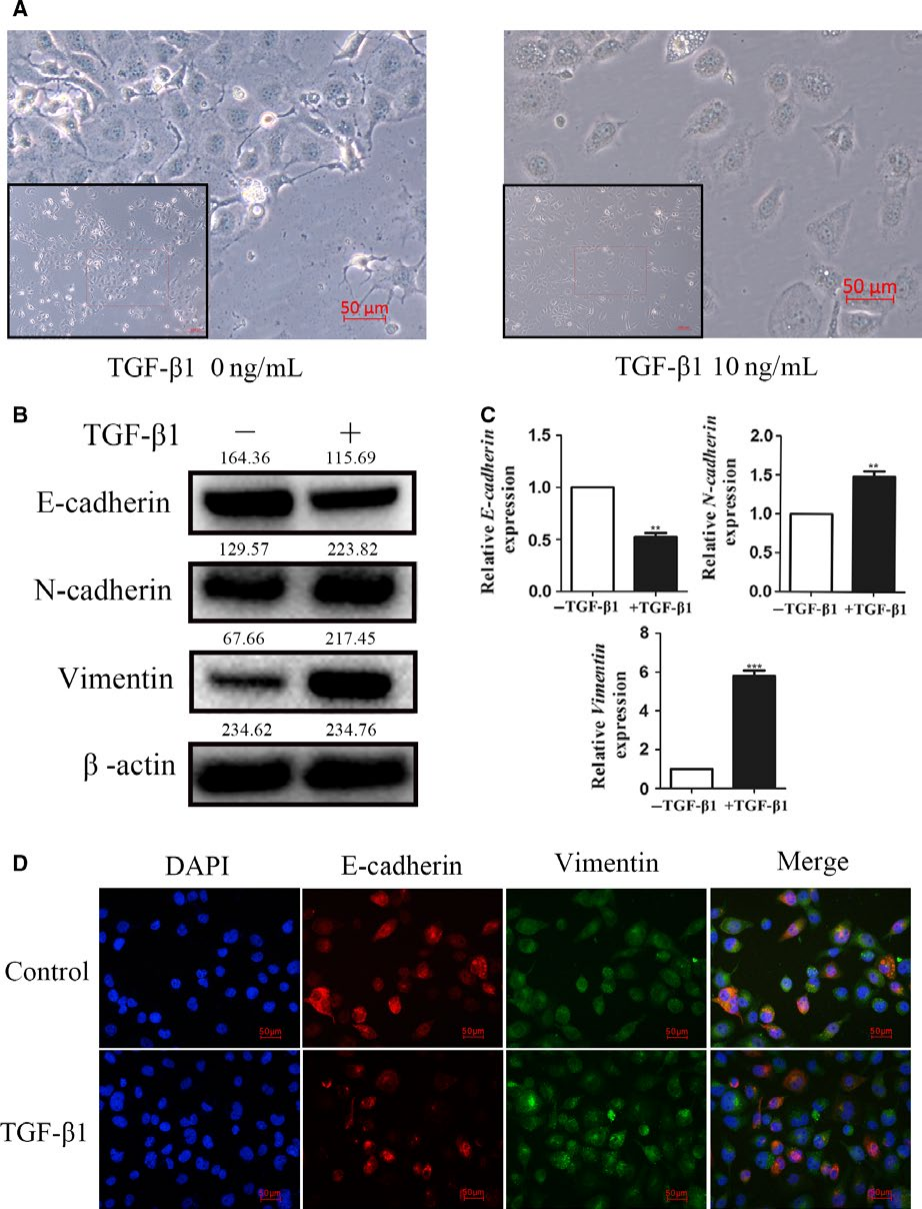
TGF‐β induces EMT in ESCC cells. A, TGF‐β‐induced cell morphological changes in EC‐1 cells. B, Western blot analysis showed the protein levels of E‐cadherin, N‐cadherin, and vimentin in EC‐1 cells treated with TGF‐β. C, qRT‐PCR showed the mRNA levels of E‐cadherin, N‐cadherin, and vimentin in EC‐1 cells treated with TGF‐β. D, Immunofluorescence analyses of EMT markers in EC‐1 cells. ***P < 0.001, **P < 0.01. TGF‐β**,** transforming growth factor‐β; ESCC, esophageal squamous cell carcinoma; EMT, epithelial‐mesenchymal transition

We used TGF‐β to incessantly stimulate the ESCC cells lines (EC‐1) firstly in order to implement the TGF‐β‐induced EMT model. Interestingly, the distance of EC‐1 cell treated with TGF‐β was significantly higher than the untreated cells. These morphological changes were observed by phase microscopy (Figure 1A). We also used western blot analysis and qRT‐PCR to investigate the expression levels of epithelial markers such as E‐cadherin, mesenchymal markers, N‐cadherin, and vimentin. Consistent with the morphological changes, after TGF‐β1 treatment for 48 h, the results presented that E‐cadherin expression was meaningfully decreased in EC‐1 cells, and N‐cadherin and vimentin were increased. (Figure 1B,C). Immunofluorescence analysis also showed that after TGF‐β1 induction, E‐cadherin expression was decreased and N‐cadherin and vimentin expressions were increased (Figure 1D). Our study clearly showed that TGF‐β1 was able to encourage EMT changes in EC‐1 cells.

### 3.2 Knockdown of SMAD4 partially reverses invasion and migration of ESCC cells induced by TGF‐β

**Figure 2:**
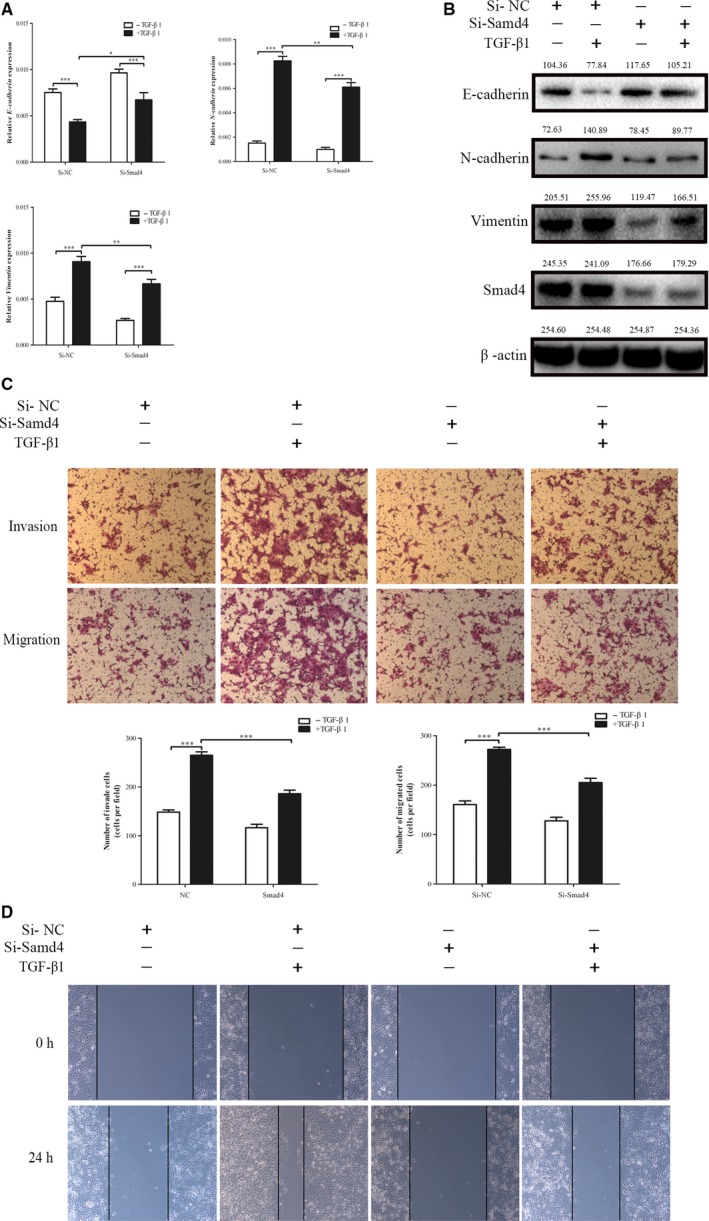
Silencing SMAD4 inhibits TGF‐β‐induced EMT of ESCC cells. A and B, SMAD4‐silenced EC‐1 cells were serum‐starved for 24 h, and treated with or without TGF‐β1 (10 ng/mL) for 48 h. Then, EMT marker mRNA and protein levels were determined using qRT‐PCR and western blot analyses. C and D, Silencing SMAD4 significantly inhibited the invasion and migration induced by TGF‐β, as determined by wound healing assays and Transwell assay in NPC cell lines. ***P < 0.001, **P < 0.01. TGF‐β**,** transforming growth factor‐β; ESCC, esophageal squamous cell carcinoma; EMT, epithelial‐mesenchymal transition

It has been reported that SMAD4 plays an important role in the progression of TGF‐β‐induced EMT.^25^, ^26^ Nevertheless, few studies have reported on the role of SMAD4 in TGF‐β‐induced ESCC. In our study, SMAD4 was knocked down by precise SiRNA against SMAD4 (Si‐SMAD4) in the EC‐1 cells. And we found that knockdown Smad4 could partially reverse the decrease of E‐cadherin expression and the increase of N‐cadherin and vimentin expression induced by TGF‐β. (Figure 2A,B). Transwell assays and wound healing further indicated that the knockdown of SMAD4 could inhibit TGF‐β‐induced migratory and invasive capabilities in EC‐1 cells at the same time (Figure 2C,D).

### 3.3 miR‐130a‐3p directly targets SMAD4 in EC‐1 cell

**Figure 3:**
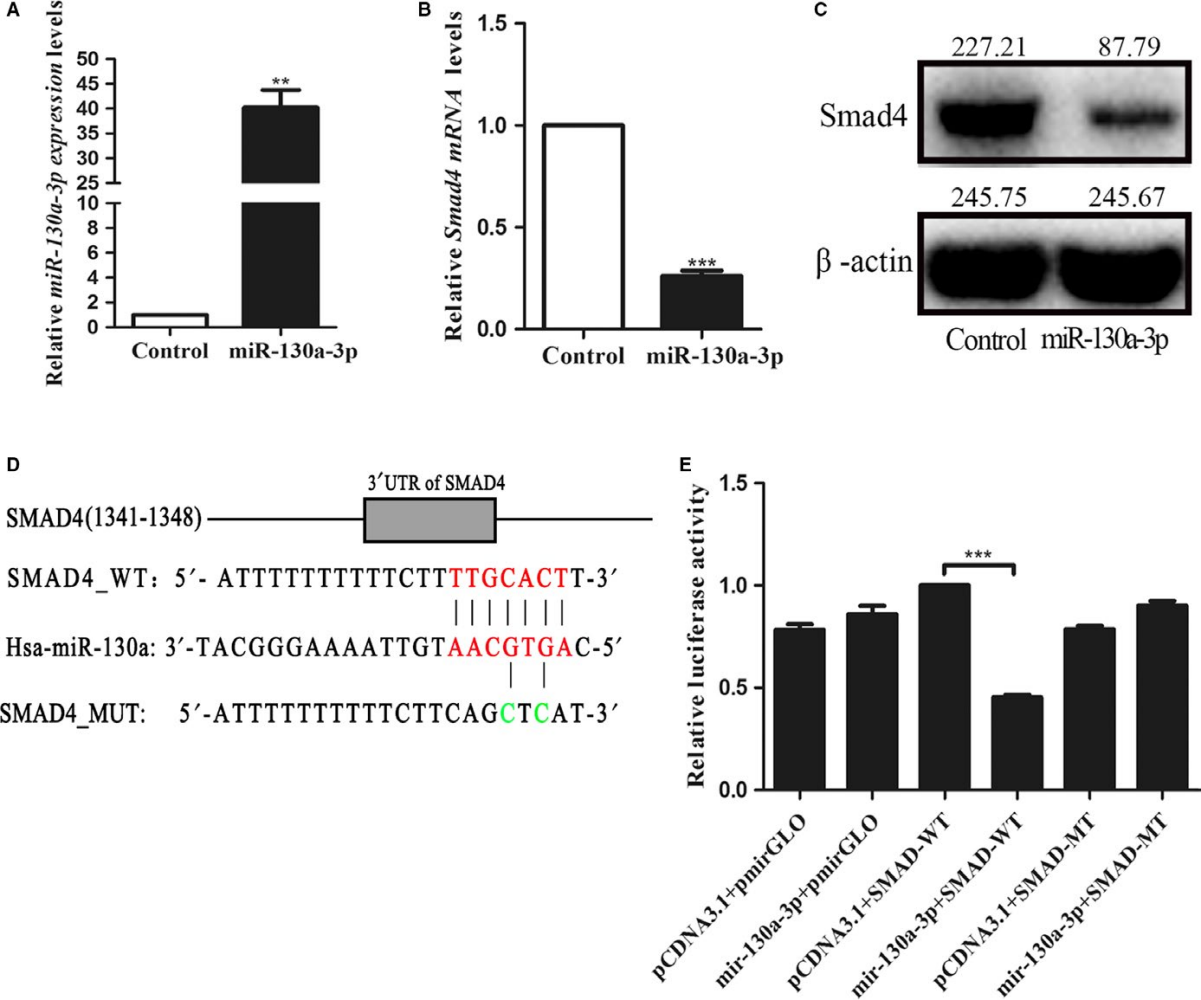
miR‐130a‐3p directly targets SMAD4 in EC‐1 cells. A, Relative real‐time PCR was used to detect the expression miR‐130a‐3p in the EC‐1 cell lines transfected with miR‐130a‐3p mimic. B and C, Quantification of SMAD4 mRNA level by qRT‐PCR (B) and SMAD4 protein level by western blot (C) after transfection with miR‐34a mimic. D, Sequences of the predicted miR‐130a‐3p binding site in 3′‐UTR of SMAD4 were shown as a schematic drawing. E, miR‐130a‐3p significantly reduced luciferase activity of the wild‐type but not the mutant 3′‐UTR of SMAD4. ***P < 0.001, **P < 0.01

Previous examination also presented that miR‐130a‐3p is related to SMAD4. To verify the function of miR‐130a‐3p in ESCC cells, miR‐130a‐3p mimic was transfected into the EC‐1 cell and then the endogenous level of miR‐130a‐3p was altered (Figure 3A). We transiently transfected miR‐130a‐3p mimic into EC‐1 cell lines and evaluated SMAD4 expression levels by qRT‐PCR and western blot analysis. The results illustrated that miR‐130a‐3p considerably decreased SMAD4 expression (Figure 3B,C). To identify our hypothesis further, a luciferase reporter assay was performed. As clearly demonstrated in Figure 3D, the cells transfected with wtSMAD4 3'‐UTR vector covering a precursor miR‐130a‐3p revealed a lower luciferase activity than the cells transfected with miR‐control (*P* < 0.05). On the contrary，we did not observe any change in relative luciferase activity with the mutated binding site of miR‐130a‐3p (Figure 3D,E). In conclusion, our results indicate that miR‐130a‐3p can directly targets the 3'‐UTR of SMAD4 in ESCC cells.

### 3.4 miR‐130a‐3p can inhibit tumor metastasis in vivo

**Figure 4:**
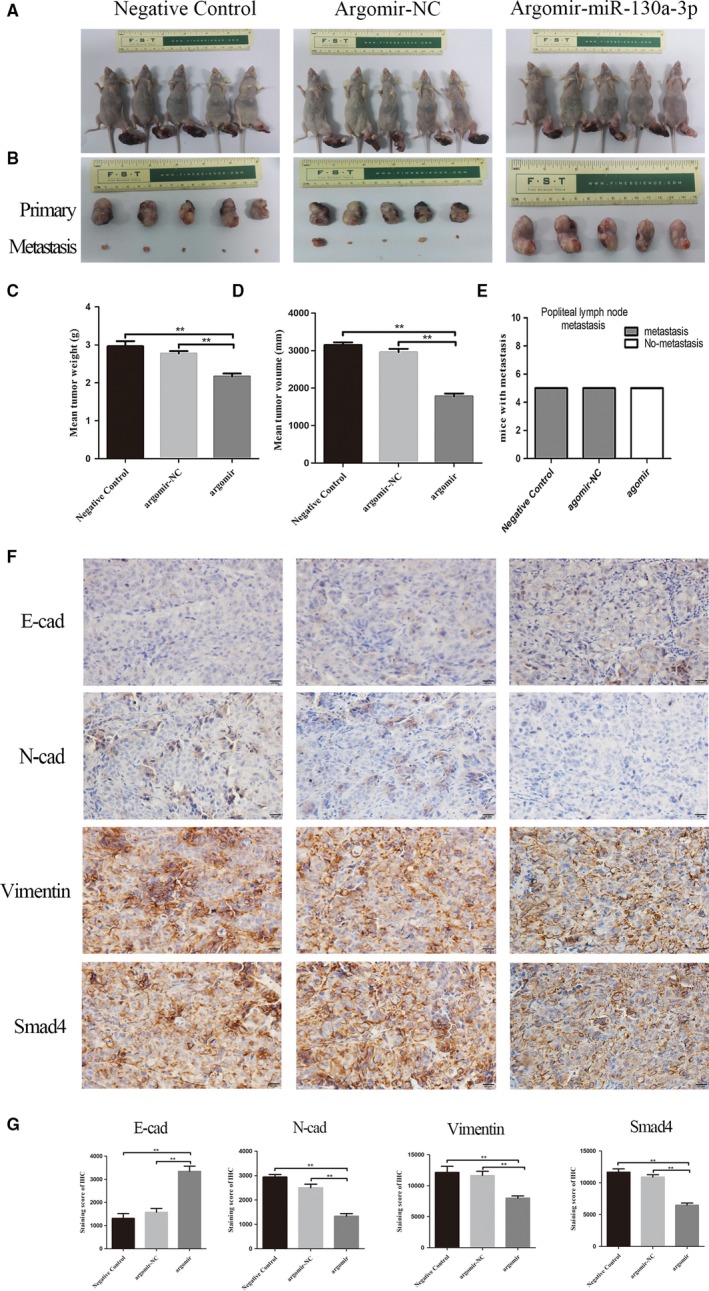
miR‐130a‐3p can inhibit tumor metastasis in vivo. A‐E, miR‐130a‐3p overexpression reduced the size of the primary tumor and tumor axillary lymph node metastasis. F and G, Immunohistochemical analysis was used to detect. Endogenous miR‐130a‐3p caused a corresponding change of EMT markers in vivo. ***P < 0.001, **P < 0.01. EMT, epithelial‐mesenchymal transition

To investigate the effect of miR‐130a‐3p on ESCC metastasis in vivo, EC‐1 cells (2 × 10^6^ cells) were injected into the sole of the nude mice. When the xenograft volume reached 60 mm^3^, the corresponding reagent was injected subcutaneously into the foot mass. The mice were euthanized 49 days later. We removed the tumors and weighed them to observe the tumor axillary lymph node metastasis. The results exposed that miR‐130a‐3p overexpression pointedly reduced the size of the tumor on the sole of the foot compared with argomir‐NC and NCs (Figure 4A). Similarly, tumor weight and volume showed the same results as size (Figure 4C‐E). Next, we examined the consequence of miR‐130a‐3p on tumor metastasis in vivo. The experimental results fully demonstrated that miR‐130a‐3p greatly inhibits tumor axillary lymph node metastasis (Figure 4B). Immunohistochemical analysis also revealed that endogenous miR‐130a‐3p caused a corresponding change of EMT markers in vivo (Figure 4F,G). In conclusion, these results designated that miR‐130a‐3p inhibits tumor metastasis in vivo.

### 3.5 Altered expression of miR‐130a‐3p was induced by TGF‐β1 in EC‐1. miR‐130a‐3p regulates the EMT, invasion, and migration induced by TGF‐β in EC‐1

**Figure 5:**
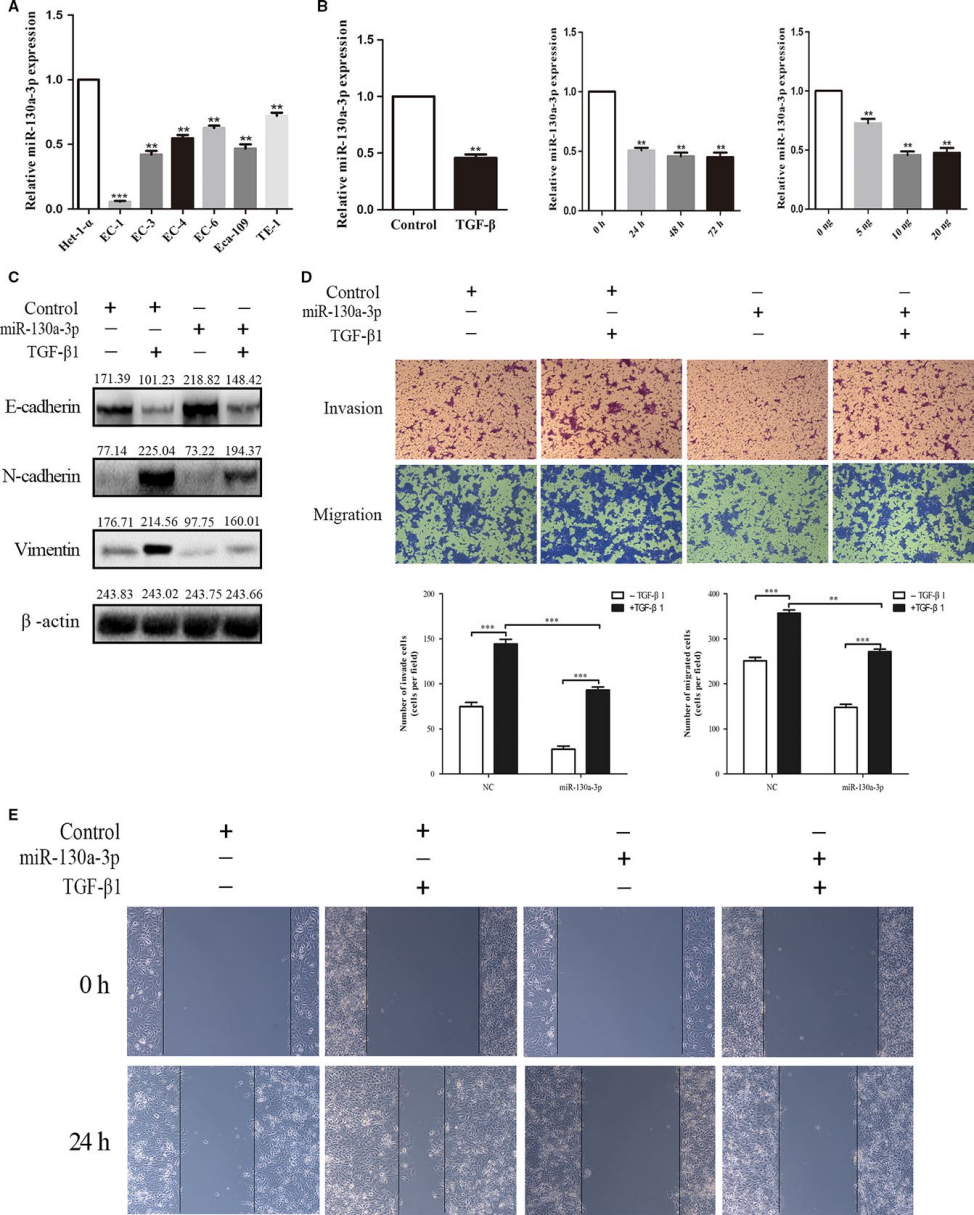
The altered expression of miR‐130a‐3p was induced by TGF‐β1 in EC‐1, miR‐130a‐3p moderates the EMT, invasion, and migration induced by TGF‐β in EC‐1. A, Expression level of miR‐130a‐3p in EC‐1 cell lines using qRT‐PCR. B, TGF‐β induced miR‐130a‐3p expression decreased. C, The restoration of miR‐130a‐3p expression decreased the protein levels of N‐cadherin and vimentin and increased the protein level of E‐cadherin. D and E, miR‐130a‐3p significantly inhibited the invasion and migration induced by TGF‐β, as determined by wound healing assays and Transwell assay in ESCC cell lines. ***P < 0.001, **P < 0.01. TGF‐β**,** transforming growth factor‐β; ESCC, esophageal squamous cell carcinoma. EMT, epithelial‐mesenchymal transition

To explore the role of miR‐130a‐3p in esophageal cancer metastasis, we determined miR‐130a‐3p expression levels in esophageal cancer cell lines (EC‐1, EC‐3, EC‐4, EC‐6, ECa‐109, TE‐1) and normal esophageal epithelial cell line (Het‐1‐A) by qRT‐PCR. Compared with Het‐1A cells, miR‐130a‐3p is significantly under expressed in all tumor cell lines (Figure 5A). To determine the involvement of TGF‐β1 on miR‐130a‐3p, we observed miR‐130a‐3p levels at different concentrations of TGF‐β1 in EC‐1. RT‐qPCR validation revealed that TGF‐β1 induced a decrease in miR‐130a‐3p expression levels in a dose‐dependent (0, 5, 10, and 20 ng/mL) and time‐dependent (0, 24, 48, and 72 hours). Meanwhile, the control (0 ng/mL) had no significant change (Figure 5B‐D). Then, we determined whether miR‐130a‐3p inhibits TGF‐β/SMAD4‐induced EMT in EC‐1 cell lines treated with TGF‐β1. In this research, TGF‐β1‐treated cells in combination with miR‐130a‐3p mimic showed higher expression of E‐cadherin and lower expression of N‐cadherin and vimentin at the mRNA level compared with mimic‐NC (Figure 5E‐H). We further used wounding healing assays and the Transwell assay to examine whether miR‐130a‐3p affects TGF‐β‐induced migration and invasion in EC‐1 cell lines. Overall, the consequences designated that the invasion and migration capacity induced by TGF‐β/SMAD4 signal pathway of ESCC cell lines can be reversed by miR‐130a‐3p.

### 3.6 Restoring SMAD4 expression rescues miR‐130a‐3p‐suppressed EMT, invasion, and migration

**Figure 6:**
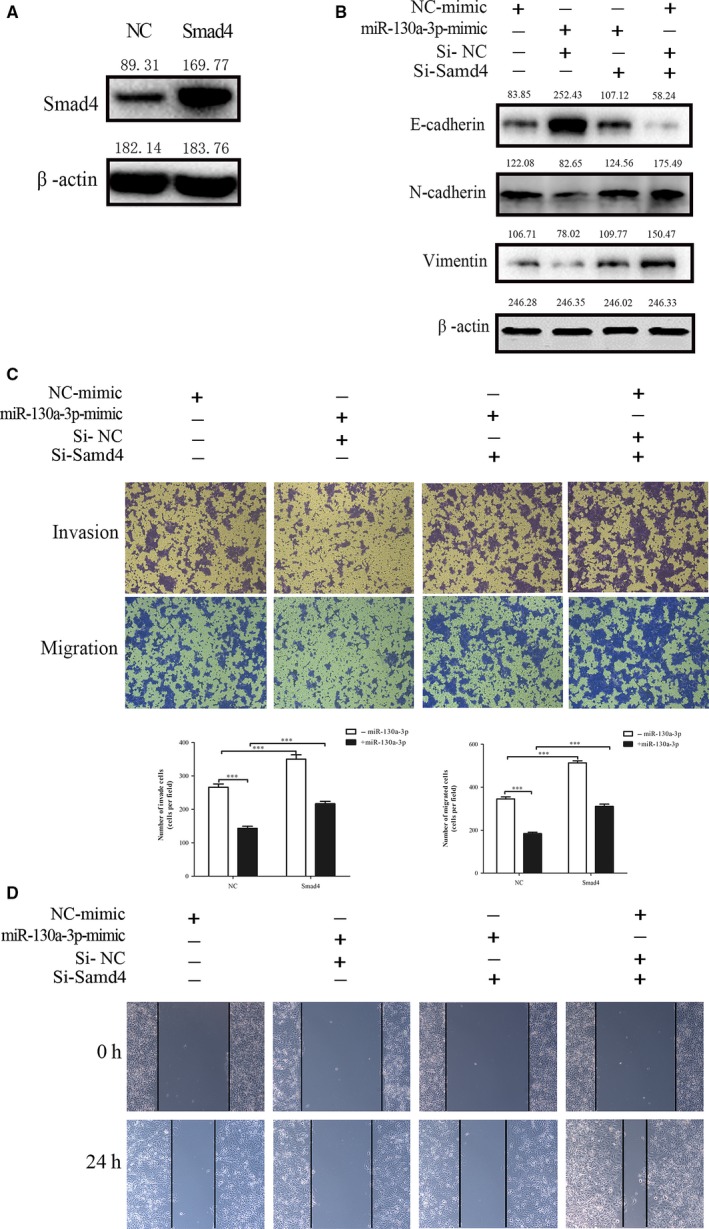
Restoring expression of SMAD4 rescues miR‐130a‐3p suppressed EMT, invasion, and migration. A, The efficacy of overexpression SMAD4 was determined by western blotting analysis. B, Restoring expression of SMAD4 antagonized the miR‐130a‐3p suppressed EMT‐related markers (N‐cadherin and vimentin). C, The invasive and migratory capacities of ESCC cell lines inhibited by miR‐130a‐3p were partially recovered by SMAD4, as determined by wound healing assays and Transwell assay. ***P < 0.001, **P < 0.01. ESCC, esophageal squamous cell carcinoma; EMT, epithelial‐mesenchymal transition

To support that SMAD4 intercedes the part of miR‐130a‐3p in TGF‐β‐induced answers in ESCC cells, we transfected pcDNA3.1‐SMAD4 plasmid into EC‐1 cells with TGF‐β stimulation as well as miR‐130a‐3p overexpression. The data showed that the restoring expression competence of SMAD4 was estimated at the protein level (Figure 6A). Furthermore, the results of western blot also showed that SMAD4 restoration in the miR‐130a‐3p overexpression group incompletely reversed the inhibitory effect of miR‐130a‐3p on EMT behaviors (Figure 6B). Furthermore, miR‐130a‐3p, which directly targeting SMAD4, could reduce TGF‐β‐induced invasion and migration depended by SMAD4. SMAD4 overexpression could also partially reverse the function of miR‐130a‐3p (Figure 6C,D). These results revealed that restored SMAD4 expression attenuates miR‐130a‐3p functions in EC‐1 cells.

## 4 DISCUSSION

Distant metastasis and recurrence remain the main causes of poor prognosis in ESCC patients. The EMT process is closely related to these malignant biological behaviors. The TGF‐β signaling pathway affects a variety of cellular events, such as cell proliferation, invasion, and migration, Therefore, it is extremely important to discover the deep mechanism of recurrence and metastasis in esophageal cancer.

TGF‐β signaling is known to induce EMT and is associated with the progression of numerous tumors.^27^ The activation of the TGF‐β/Smads signaling pathway is started by TGF‐β binding to ligands. TGF‐β first binds to TβRII on the cell membrane surface to form a heterodimeric complex; and TβRII rephosphorylates the serine/threonine of TβRI. The acid residues cause the ligand, TβRI, and TβRII to form a receptor complex, and the activated TβRI further phosphorylates the downstream R‐Smads, namely Smad2 and Smad3. Then, the R‐Smads and Co‐Smads, SMAD4, formed a heterodimeric complex that is transferred into the nucleus and, together with other transcription factors, regulated the expression of the target gene.^28^ Qiao et al^29^ informed that miR‐34a repressed EMT in human cholangiocarcinoma by directing SMAD4 through the TGF‐β/Smad signaling pathway. Zeng et al^13^ reported the suppression of SMAD4 by miR‐205 regulates TGF‐β‐induced EMT in Squamous cell carcinoma cell lines. In our experiments, we first introduced TGF‐β1 exogenously to induce EMT progression in cells. In our induction model, the cell morphology changed, cell adhesion decreased, cells lost contact, while the appearance of the epithelial marker E‐cadherin decreased, the interstitial markers N‐Cadherin and vimentin increased.

The common‐medium SMAD (Co‐SMAD) only includes SMAD4, and SMAD4 is an important central medium for the TGF‐β signaling pathway. The deletion of SMAD4 in patients with pancreatic cancer revealed that SMAD4 deletion or mutation may promote the progression of pancreatic cancer.^30^ Researchers found that SMAD4 plays a tumor suppressor role in many cancers, such as gastric cancer (GC),^26^ colorectal cancer,^25^ and cholangiocarcinoma.^29^ However, a large number of research showed that SMAD4, a significant factor in the TGF‐β/SMAD4 signaling pathway, plays a crucial part in signaling into the cell membrane and can contribute to the progression of EMT,^9^, ^14^, ^31^ Unfortunately, The specific role of SMAD4 in TGF‐β‐induced EMT in ESCC remains understudied. We provided evidence that knocking down of SMAD4 expression could partially reverse TGF‐induced EMT progression, increases E‐Cadherin protein expression, decreases N‐Cadherin and vimentin protein expression, and inhibits the invasion and migration of EC‐1 cells.

It is well‐known that miRNAs are involved in the progression of EMT in cancer by directly regulating the expression of their specific target at the posttranscriptional level. It has been indicated that miRNAs play an irreplaceable part in TGF‐β‐induced EMT.^13^, ^32^, ^33^ Mengtao Ma et al found that MAGI2 promotes TGF‐β1‐induced EMT and migration and invasion of breast cancer cells under the direct action of miR‐487a.^34^ miR‐199b‐5p promotes cell aggregation, suppresses cell invasion and migration and inhibits TGF‐β‐induced EMT in hepatocellular carcinoma by inhibiting N‐cadherin.^35^ In several studies, miR‐130a‐3p was shown to decrease in cancer tissues and inhibit cell invasion, migration, and EMT.^36^, ^37^, ^38^ By contrast, miR‐130a‐3p is upregulated in some GC cases and promotes cell proliferation and migration in GC cells. In our study, we confirmed that miR‐130a‐3p was greatly downregulated in esophageal cancer cell lines compared with normal epithelial cells Het‐1A. Additionally, we found that miR‐130‐3p suppresses the esophageal cancer metastasis in vitro and in vivo. More importantly, the exogenous expression of SMAD4 partially recovered the invasive and migratory ability of EC‐1 cell lines, which was repressed by the overexpression of miR‐130a‐3p. The overexpressed SMAD4 similarly rescues the EMT progression even in the existence of miR‐130a‐3p.

In conclusion, we demonstrated that miR‐130a‐3p is a tumor suppressor in esophageal cancer. Based on the findings from this study and others, we verified that miR‐130a‐3p suppresses the function of TGF‐β/SMAD4 signal‐induced EMT and the invasion and migration in EC‐1 cell lines depended on SMAD4.

## CONFLICT OF INTEREST

None declared.
